# Dissolution Processes of PFSA Polymers via Mixed Solvents and Their Effects on Structural, Morphological and Electrochemical Activity

**DOI:** 10.3390/molecules31111856

**Published:** 2026-05-28

**Authors:** Mveliso Ester Hlwele, Opeoluwa O. Oyedeji, Edson L. Meyer, Nicholas Rono, Mojeed A. Agoro

**Affiliations:** 1 Fort Hare Institute of Technology, University of Fort Hare, Private Bag X1314, Alice 5700, Eastern Cape, South Africa; 224085279@ufh.ac.za (M.E.H.); emeyer@ufh.ac.za (E.L.M.); nrono@ufh.ac.za (N.R.); 2 Department of Chemistry, University of Fort Hare, Private Bag X1314, Alice 5700, Eastern Cape, South Africa; ooyedeji@ufh.ac.za; 3 Department of Basic Sciences, Tharaka University, Marimanti P.O. Box 193-60215, Kenya

**Keywords:** membranes, polymer, dissolution, mixed solvents, morphology, electrochemical

## Abstract

Proton exchange membrane fuel cells (PEMFCs) exhibit high energy efficiency and rapid load response, but challenges are faced in membrane fabrication, including the need for renewable resources and cost-effective, non-toxic solvents. This study analyzes the morphological and structural properties of perfluorosulfonic acid (PFSA) ionomer membranes, FS-930 and F-14100, after the dissolution of membranes via ratios of 50:50, 80:20, and 20:80 by volume of dimethyl sulfoxide (DMSO) and water. Bode plot analysis indicates that membranes rich in DMSO show lower frequency phase angle peaks, suggesting better segmental motion and ionic conductivity. Additionally, higher DMSO content correlates with broader FTIR peaks, reflecting enhanced solute–solvent interactions. The untreated FS-930 membrane demonstrates significant intensity peaks linked to semi-crystalline domains, indicating strong baseline conductivity. SEM analysis revealed surface roughness variations in FS-930 linked to different water-to-DMSO volume ratios. DMSO-rich mixtures produced dense, hydrophobic PFSA membrane structures, whereas water-rich mixtures increased water uptake and ionic conductivity. Fumapem F-14100 showed superior hydration and proton conductivity compared to FS-930 because it contains more sulfonic acid groups. These findings are critical to understanding how membrane properties relate to solvent composition, aiding in the optimization of membrane fabrication for better performance and durability in fuel cells.

## 1. Introduction

Membrane technology has become more significant due to its increased efficiency and low cost, as well as its worldwide range of applications in different fields such as energy conversion and storage, environmental protection and remedy, substance separation, and purification [1,2,3,4,5]. Depending on the material used in synthesis, membranes can be categorized as either inorganic or organic. In contrast to inorganic membranes (which are made of materials like ceramics, carbon molecular sieves, zeolites, and amorphous silica), organic membranes are made of petroleum-derived synthetic polymers like polysulfone (PS), polytetrafluoroethylene (PTFE), polyethersulfone (PES), and polyvinylidene fluoride (PVDF) [6,7]. The ability of polymeric membranes to provide selective permeability makes it possible to effectively separate different components from complex mixtures valuable [8,9]. Their versatility allows them to be used in a variety of industries, including desalination, water treatment, food processing, and even pharmaceutical applications. Understanding the dissolution process facilitates the optimization of design and processing conditions, as well as the choice of an appropriate solvent [10,11]. Polymer dissolution is crucial in various industrial applications, particularly in membrane fabrication where different organic solvents are utilized for this process [12]. Traditional solvents used in membrane synthesis, such as dimethylformamide (DMF), dimethylacetamide (DMAc), and dimethyl sulfoxide (DMSO), water, and ethanol, are recognized as toxic [13]. Appropriate solvent selection is crucial for effective polymer dissolution, significantly influencing membrane research, especially in creating asymmetric membranes via phase inversion. This process involves casting a polymer solution onto a substrate and solidifying it in a coagulation bath [14,15].

**Scheme 1:**
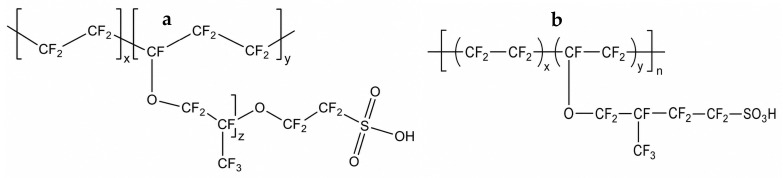
Chemical structure of perfluorosulfonic acid (PFSA) ionomer structure illustrating the backbone and sulfonated side chain typical of F-14100 (**a**) and FS-930 (**b**) membranes [17].

The final structure of fluoropolymer membranes depends on the degree of polymer dissolution. These membranes are composed of synthetic polymers containing fluorine, including (i) polymers with fluorinated dangling chains such as poly[fluoro(oxetane)] fluorinated polyurethanes, and poly[fluoro(meth)acrylate] (ii) perfluoropolyethers (PFPEs) created by combining hexafluoropropylene (HFP) and tetrafluoroethylene (TFE) with oxygen or through ring-opening polymerization of oxetanes or hexafluoropropylene oxide (HFPO), and (iii) polymers with fluorine and carbon in their backbones. Fluoropolymers are known for their exceptional non-stick properties, hydrophobic nature, low friction, high thermal stability, and chemical resistance (Souzy and Ameduri, 2005) [16]. These appealing characteristics render them suitable for numerous applications, especially in severe conditions where alternative materials might fail [16]. Membranes are effective in various aqueous applications, such as haemodialysis, oil–water separation, seawater desalination, and wastewater treatment from both urban and industrial sources [17]. Fluoropolymer membranes like Fumapem (F-14100) and FS-930 are critical for controlled release and removal of toxic substances in separation processes. Fumapem (F-14100), a high-performance ion exchange membrane from FUMATECH BWT GmbH (Bietigheim-Bissingen, Germany), is known for its robust chemical resistance and thermal stability, suitable for harsh filtration environments. Composed of tetrafluoroethylene (TFE) and vinylidene fluoride (VDF), it offers low surface energy and excellent mechanical qualities. This 100 µm thick membrane displays remarkable stability in acidic conditions, low resistance, and minimal dimensional swelling, paralleling the properties of Nafion [18] due to its crystalline nature and high degree of fluorination thus, challenging to dissolve using traditional solvents. Scheme 1 displays the chemical structure of perfluorosulfonic acid (PFSA).

In FS-930 and F-14100 PFSA ionomer membranes, the electrochemical mechanisms are governed by the transport of protons and dissociation through hydrated sulfonic acid groups (–SO_3_H) attached to a hydrophobic polytetrafluoroethylene (PTFE) backbone. The dissociation of the –SO_3_H terminal bond in the presence of water gives rise to mobile protons (H^+^) and fixed sulfonate ions (–SO_3_^−^). Water absorbed molecules form a network of nanochannels known as hydrophilic ionic within the fluorinated polymer matrix. Thereby allowing proton conduction through proton migration of hydronium ions (H_3_O^+^), known as the vehicular mechanism, while the rapid transfer of protons between sulfonic acid sites and adjacent hydrogen-bonded water molecules occurs due to the Grotthuss hopping mechanism. The membrane acts as a selective proton conductor and as an electronic insulator simultaneously, paving the way for H^+^ transport while reactant crossover and blocking electrons. Equivalent weight (EW) serves as the difference, as it strongly influences the electrochemical activities of these membranes. The lower sulfonic acid density and higher EW of F-14100 are associated with fewer ionic domains, reduced water uptake, enhanced mechanical, lower proton conductivity, and chemical stability. On the other hand, FS-930 has a lower EW with a higher concentration of sulfonic acid groups, giving rise to better hydration, enhanced proton conductivity, highly interconnected ionic channels, improved ion exchange capacity, and higher swelling tendency. Thus, PFSA membranes’ proton transport efficiency is primarily governed by the sulfonic acid group density, hydration level, and ionic domain morphology [19,20,21].

Fumasep FS-930 is a perfluorinated cation exchange membrane that shares dissolution characteristics with the F-14100 membrane but offers greater flexibility. Despite its compatibility with certain solvents, its molecular structure, characterized by strong carbon–fluorine bonds, complicates dissolution. Table 1 shows the physical and chemical data of the F-14100 and FS-930 cation exchange membrane. The FS-930 membrane also demonstrates resistance to solvent-induced in strong alkaline solutions [22]. FS-930 is a widely used exchange membrane (PEM) known for its excellent proton conductivity and chemical stability in electrochemical applications such as fuel cells and water electrolysis [23]. Membranes are typically pre-treated with solvents like ethanol to eliminate contaminants and then dried at 60 °C for 24 h. F-14100 and FS-930 membranes may undergo immersion in mixed solvents at varying temperatures.

**Figure 1:**
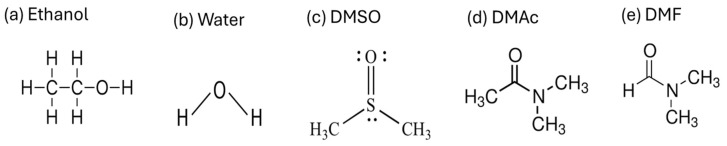
Chemical structures of the mixed solvents: (**a**) Ethanol, (**b**) Water, (**c**) Dimethyl sulfoxide (DMSO), (**d**) Dimethylacetamide (DMAc) and (**e**) N, N-Dimethylformamide (DMF).

Generally, solvents used for dissolution include DMF, DMAc, DMSO, water, and ethanol; this study mainly focuses on DMSO and water. Water causes phase separation and morphological remodeling, while DMSO encourages membrane disintegration and structural expansion; their combined action determines the ultimate membrane properties. The dissolution rates were assessed by measuring changes in membrane structure, solvent-induced radiation, and monitoring weight loss [24]. Figure 1 below shows the different types of traditional solvents used for dissolution of fluoropolymer membranes.

Various experimental methods that may be utilized to characterize the dissolution products of polymers include differential refractometry (concentration of solutes), fluorescence microscopy (interactions and dynamics of membrane molecules), gravimetry (membrane casting processes), interferometry (thickness), nuclear magnetic resonance (identifying the chemical compounds), and Fourier transform–infrared (functional groups) [25]. Dissolution alters the structural and morphological properties of membranes, with mixed solvents reducing their crystallinity and interfering with polymer chains, thereby affecting their mechanical and surface properties [26]. Membrane dissolution is essential for membrane recycling, the creation of a functionalized layers of filtration process and reprocessing [27]. Proton exchange membrane fuel cells (PEMFCs) are an efficient power source suitable for portable, motor, and stationary applications; this is attributed to their low operating temperature, rapid load response, minimal emissions, and high energy conversion efficiency [28]. This study aims to enhance our understanding of membrane technology while also encouraging a wider conversation about the role of innovative materials in advancing sustainable energy in the future [29]. The dissolution behavior of F-14100 and FS-930 fluoropolymer membranes in mixed solvent systems of water and DMSO at different ratios (20:80, 50:50, and 80:20) was examined in this study, to assess how these solvent ratios affected the membranes’ morphological and structural characteristics and to identify solvent compositions that optimize ionic conductivity performance.

Various characterization tools can assess physical and chemical transformations after dissolution, observe morphological changes, and examine changes in chemical structure. The result of this work aims to provide insights into the use of mixed solvents and the complications that arise when using mixed solvent systems, paving the way for an accurate evaluation of solvent ratio–membrane interactions. Understanding these solvent ratio interactions changes, as reported in this study, enhances our understanding of advancing membrane technology and its application in fuel cells.

## 2. Results and Discussions

### 2.1. The Chemical Structure of the Membrane Samples

**Figure 2:**
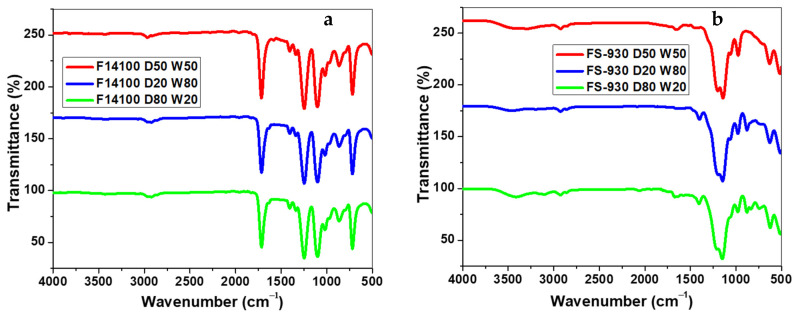
FTIR Spectrum of F-14100 (**a**) and FS-930 (**b**) membrane.

The hydration and dehydration behavior of materials produced as thin films and freestanding membranes was investigated using FTIR. Figure 2a presents an FTIR spectrum of the F-14100 membrane dissolved in various water and dimethyl sulfoxide (DMSO) ratios: 20 mL H_2_O to 80 mL DMSO, 80 mL H_2_O to 20 mL DMSO, and a 50:50 ratio. These spectra reveal significant differences in the chemical interactions and structural integrity of the membrane depending on the solvent ratio used. Ionizable functional groups found in polymers serve as the building blocks for materials that facilitate selective ion transport in a variety of real-world uses, such as the production of electricity, chemical separations, and water purification [30]. The molecular environment and interactions between the solvent and the membrane are significantly altered after dissolution, as shown in the FTIR spectrum. Figure 2b illustrates the bending modes of water molecules with H–O–H bending vibrations at about 1600–1660 cm^−1^. Sulfonic acid S=O was observed at around 1220–1260 cm^−1^, while the polymer backbone was shown by C–F and CF_2_ backbone vibrations at about 1200–1140 cm^−1^ [31]. The increasing intensity indicates a significant intake of water. The FTIR spectrum of FS-930 following its dissolution in mixed solvents by volume ratios is displayed in Figure 2 below.

Figure 2b presents the FTIR spectrum of the FS-930 membrane, highlighting broad O–H stretching vibrations near 3500 cm^−1^, indicative of water presence. The higher presence of OH stretching observed in D50-W50% is linked to distinct, strong DMSO–water intermolecular interaction between water and sulfonic acid groups –SO_3_H, leading to intermediate complexes retention of water H_3_O^+^-DMSO, which effectively alters the OH groups concentration order. Figure 2a exhibited more pronounced bands due to an 80 mL (80%) H_2_O ratio in the membrane dissolution. A C-H stretch related to methyl groups appears around 3000 cm^−1^, alongside a significant broad sulfonic acid stretch between 1200–1140 cm^−1^. The intensity increase of these bands suggests the membrane’s high hydrophilicity. Spectral subtraction is utilized to distinguish spectral features in polymer mixes and to eliminate solvent bands or separate features following chemical or physical changes [32].

Figure 2a,b reveal the interactions between membranes and mixed solvents. The F-14100 membrane shows more distinct and wider peaks upon dissolution compared to the FS-930 membrane, indicating pronounced changes in molecular interactions. Higher DMSO content leads to broader FTIR peaks and altered band positions, signifying enhanced solute-solvent interactions. In a water-rich (80:20 H_2_O: DMSO) mixture, F-14100 retains sharp solid-state bands, suggesting limited solubility, whereas DMSO-rich mixtures exhibit weaker and broader bands, indicating better solvation and potential hydrogen-bond formation with DMSO’s S=O group. DMSO, as a polar aprotic solvent, effectively disrupts solute interactions, promoting better dissolution of the more hydrophobic F-14100. Retained spectrum characteristics and morphological integrity show that the membranes experience swelling and partial structural disruption rather than total breakdown.

### 2.2. Raman Spectroscopy Analysis

#### 2.2.1. Vibrational Modes Characterization of Sample After Dissolution

**Figure 3:**
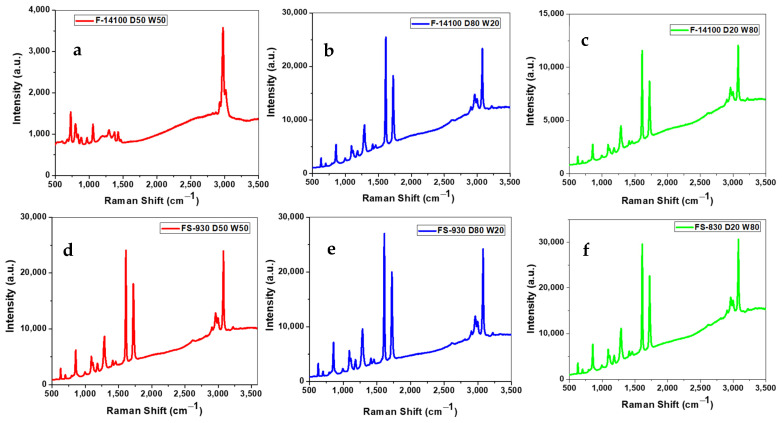
Raman Spectra of F-14100 (**a**–**c**) and FS-930 (**d**–**f**).

In this work, confocal Raman microscopy (CRM) was used to investigate application-relevant properties of perfluorinated sulfonic acid (PFSA) membranes. Peak intensity variations can provide essential information regarding the mechanical and structural qualities of the membrane [33]. Figure 2a displays the Raman spectra of the F-14100 membrane, highlighting an O-H stretching around 3200 cm^−1^ indicative of hydration during dissolution. Figure 3d–f show the FS-930 membrane’s Raman spectra following dissolution with H_2_O and DMSO in varying ratios. The Raman peaks at 626–634 cm^−1^ were ascribed to backbone C–H rocking and wagging modes. Symmetrical bond resonance of SO_3_H is observed at approximately 1061–1090 cm^−1^; however, this peak had low intensity in some samples, which indicates a potential decrease in SO_3_H groups on the membrane side chain. Spectral differences are also evident, with noticeable peaks at 1284 cm^−1^ and 1181–1198 cm^−1^ associated with symmetrical bond stretching of the νa(C–F) bb and νs(C–F) bb bonds from the polytetrafluoroethylene (PTFE) backbone of the FS-930 and F-14100 ion exchange membranes. The spectral shift toward the C–F backbone mode was influenced by the molecular environment of the vs(C–F)_sc_ stretching, aligning more with the PTFEbackbone structure than that of Nafion, given that both membranes belong to the Nafion family. These peaks are in good agreement with the literature by [34]. The spectral positions of the side chain-related and main backbone Raman modes for FS-930 and F-14100 membranes are derived from the Raman spectrum and presented in Table 1.

Figure 3 Shows images with weak C–H stretch at around 3000 cm^−1^ and C-F stretch at about around 1000–1200 cm^−1^, a strong C–C stretch at around 1400–1600 cm^−1^ and Strong O–H stretching at about 3000 cm^−1^. The Raman spectra of FS-930 and F-14100 demonstrate similar solvation trends influenced by DMSO content, akin to FTIR data. Regarding the 80:20 H_2_O and DMSO mixtures, both compounds showed solid vibrational bands, indicating incomplete dissolution. Increasing DMSO leads to enhanced solid-state features and reduced relative band intensities, signifying a shift from crystalline to disordered states. Notably, solvent mixtures with high DMSO content exhibit small shifts in vibrational frequencies and increased broadening, suggesting stronger solute-solvent interactions. Higher DMSO fractions enhance dissolution, whereas water-rich mixtures maintain solid-like characteristics, with F-14100 displaying lower solubility in these conditions.

#### 2.2.2. Water Uptake Analysis

**Figure 4:**
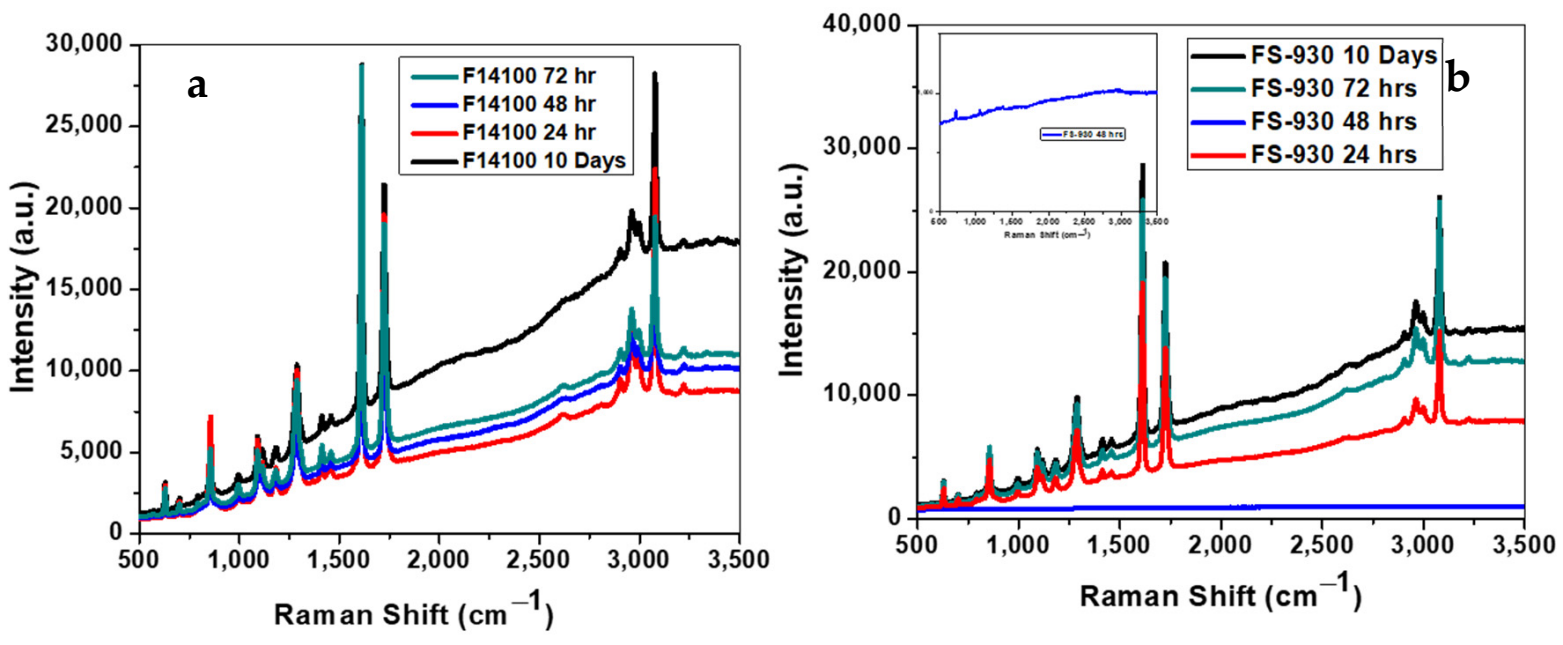
Water uptake of F-14100 (**a**) and FS-930 (**b**).

Figure 4 presents the Raman spectrum images indicating the water uptake of F-14100 (Figure 4a) and FS-930 (Figure 4b) after 24, 48, 72 h, and 10 days, to understand the effects on the membranes. By tracking vibrational shifts in the O–H stretching area and polymer functional groups, Raman spectroscopy is frequently used in membrane science to examine water intake and water–polymer interactions, offering information into hydration and structural changes inside the membrane. Proper water management is crucial for PEMFC performance, and CRM has been utilized to examine water content in PEMs both in-situ and during operation, such as studying temperature-dependent water uptake in active fuel cells [35]. Since water is typically the proton carrier and the medium for proton transfer, water uptake is crucial. The water uptake shown in Table 2 was calculated using Equation (1). Table 2 presents the results for the two membranes immersed in water for different time intervals.

(1) $$Water\;uptake\;\%=\frac{W\left(wet\right)-W(dry)}{W(dry)}\times 100$$

The water uptake of membranes, after being immersed and dried, ranged from 10.68% to 29.93%. The Raman spectra were normalized using the CF_2_ stretching vibration band at 731 cm^−1^, deemed least affected by water. Peaks associated with the Nafion backbone and side chains appeared at approximately 1211, 1065, 970, and 804 cm^−1^, while C-C single bond peaks were observed around 1297 and 1376 cm^−1^. These characteristics are consistent with previous studies on Nafion variants FS-930 and F-14100 [36]. F-14100 showed the highest uptake compared to FS-930 ionomer membranes. It increased from about 18.45% to 29.93% after 10 days, while F-14100 after 10 days increased from 10.68% to 18.02%, suggesting low absorption of water and low swelling. A higher amount of water uptake generally improves ionic conductivity since absorbed water expands, facilitating ion mobility and hydrophilic channels.

### 2.3. XRD Structural Analysis of FS-930

**Figure 5:**
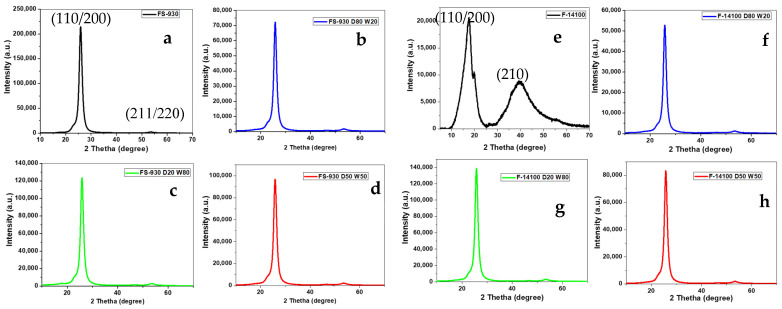
XRD diffractograms of FS-930 (**a**–**d**) and F-14100 (**e**–**h**) membrane.

X-ray diffraction (XRD) analysis was conducted to evaluate the crystallinity of membranes, with Figure 5a illustrating the original and post-dissolution patterns for FS-930.

Figure 5a–d and Table 3 present various XRD patterns of membranes before and after dissolution. Figure 5a shows untreated patterns, while Figure 5b–d,f–h illustrate FS-930 and F-14100 membrane dissolution with different H_2_O to DMSO ratios: 20 mL H_2_O/80 mL DMSO, 80 mL H_2_O/20 mL DMSO, and 50 mL of each, respectively. Figure 5b further demonstrates the dissolution of FS-930 with 20 mL H_2_O/80 mL DMSO. The peaks of both membranes correlate with amorphous carbon (JCPDS #4300180) with crystalline backbones, the F-14100 and FS-930 membranes at ~18° (110/200) and ~40° (210) (2θ) correspond to the interplanar spacing as a result of hydrogen bonding/increased chain packing [37]. The X-ray scattering from the membrane’s amorphous region at lower Bragg angles aligned with the significant peak at ~18°, linked to the crystalline scattering from the polyfluorocarbon chains in ionomer membranes [38]. The untreated F-14100 membrane exhibits a prominent intensity peak and larger crystallite sizes compared to FS-930, indicating baseline conductivity and superior mechanical order in ionomer membranes formulated from PFSA. Table 3 shows XRD parameters for F-14100 and FS-930 membranes.

Figure 5 illustrates the dissolution of F-14100 in varying solvent ratios: 20 mL of H_2_O with 80 mL of DMSO, then reversed, and finally 50 mL of each. Figure 5e–h presents the untreated F-14100 membrane. The solvent causes partial disruption of crystallographic domains, enlarges ionic clusters, and induces chain disorder in the membranes. Figure 5 indicates that the diffraction patterns reveal only minor variations in the structural features of membranes following dissolution due to the differing compositions of FS-930 and F-14100.

### 2.4. Scanning Electron Microscopy (SEM) and Energy Dispersive X-Ray Spectroscopy (EDX) Analysis

**Figure 6:**
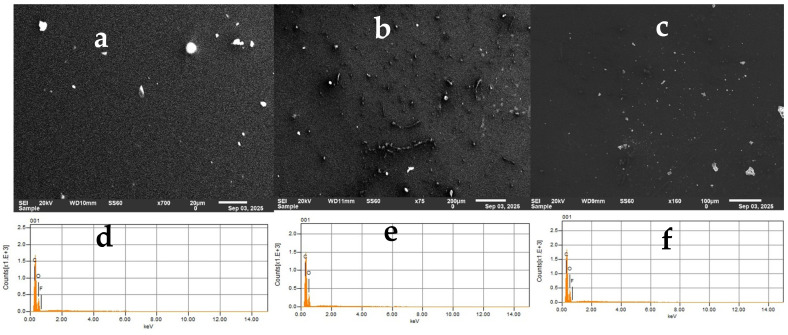
SEM (**a**–**c**) and EDX (**d**–**f**) of FS-930.

The study utilized SEM images and EDX elemental mapping to analyze the morphology and structure of FS-930 after dissolution, highlighting performance variances. The SEM analysis illustrated surface roughness changes in FS-930 due to different water to DMSO volume ratios. Additionally, the PFSA ionomer membranes FS-930 and F-14100 as seen in Figure 6a–f were characterized to assess electrode surface consistency [39]. SEM can be used to measure the pore size of a porous membrane [40]. EDX is a technique for analyzing surface composition. It was employed to assess the membranes’ major elements such as fluoride, carbon, and platinum before and after AST testing, using a PHI 5000 Scanning ESCA Microprobe [41].

**Figure 7:**
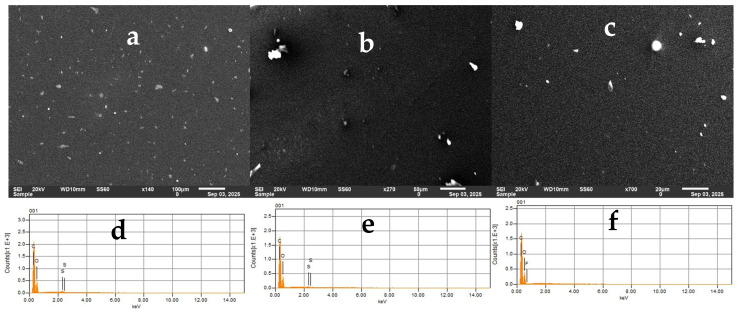
SEM (**a**–**c**) and EDX (**d**–**f**) images of F-14100.

EDX analysis of FS-930 dissolution revealed three solvent compositions: (a) 80% DMSO and 20% water, (b) 50% of both solvents, and (c) 80% water and 20% DMSO. Visible functional groups comprising carbon, oxygen, and fluorine were present across all samples, with sulfur from sulfonic acid detected only in the 80% DMSO solution. SEM and EDX imaging for the F-14100 membrane (Figure 7a–f) revealed similar solvent compositions, highlighting the structural variations in membranes post-dissolution. This could be due to sulfonic acid sites or the presence of sulfonic groups; a high background oxygen content, which enhances their detection and affects the detection of low atomic weight fluorine, or impurities based on organic carbon.

Table 4 below summarizes the membrane’s elemental composition as measured by EDX. Each sample’s primary elements are identified by the analysis, together with their corresponding weights and atomic percentages. The major elements identified by the EDX spectra of the FS-930 and F-14100 membranes were carbon, oxygen, and fluorine, which is in line with the material’s anticipated composition. A portion of the detected elements in the EDX analysis came from external sources, such as the carbon tape used to mount the samples and potential trace residues from DMSO or other organic species that remain on the membrane surface after drying. This is indicated by the carbon peak, which is indicated by an asterisk (*C), such as the other elements in the analysis. In samples made with a higher DMSO content, such as FS-930 (50:50 H_2_O:DMSO), this effect was more noticeable due to denser, smoother surfaces seen under a scanning electron microscope, which might hold onto solvent residues or impurities based on organic carbon. In F-14100, larger oxygen and fluorine peaks were seen in the water-rich membranes (80:20 H_2_O:DMSO), indicating a cleaner surface composition and more efficient solvent removal. Given the circumstances, the EDX data shows that both FS-930 and F-14100 maintain their distinctive elemental composition at various solvent ratios, and the asterisked carbon peak aids in differentiating intrinsic carbon from trace amounts of external or preparation-related carbon.

### 2.5. Atomic Force Macroscopy (AFM) Analysis

**Figure 8:**
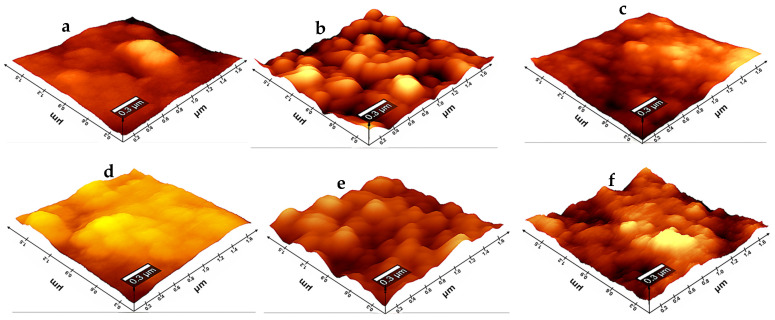
AFM 3D surface roughness of F-14100 (**a**–**c**) and FS-930 (**d**–**f**) membranes.

**Figure 9:**
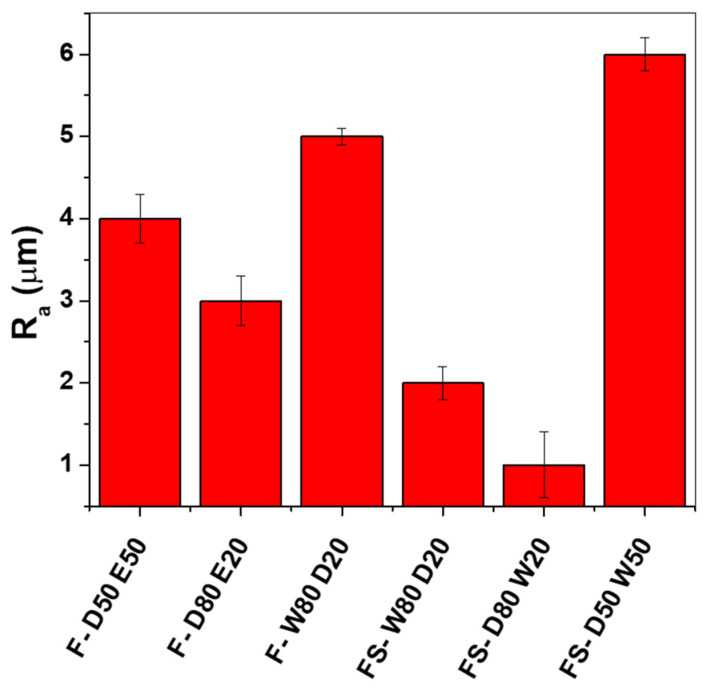
Comparison of Ra values for F-14100 as (F-) and FS-930 as (FS-) membranes.

AFM analysis was employed for F-14100 and FS-930 membranes at 80% DMSO and 20% water, 20% DMSO and 80% water and 50%. All the topographical images wereon an area of 4.0 mm^2^. The 3D surface topography in Figure 8b,e illustrates a non-smooth F-14100 and FS-930 membrane surface for 20% DMSO and 80% water, with swelling surface particles. This suggests that subsequent solvent ratio and operational conditions play a role in the structure of the membrane, particularly the ionic clusters, thereby affecting ionic transport channels. The literature [42] reports that dark regions could also suggest a membrane with a hydrophobic microphase. Comparative analysis of the Ra values of 0.084 ± 0.01 and 0.065 ± 0.02 for F-14100 and FS-930, as seen in Figure 9, suggests that, despite the derivation of both membranes from the same solvent ratio, the processes and conditions to which they were subjected resulted in noticeable alterations. Figure 8a,c,d,f show a moderately smooth surface, with a singular bright region representing the height of the membrane surface. This elevated region corresponds to surface extensions on the membrane, suggesting minor surface chippings. Such typical morphological features provide insights into the stability and surface properties, resulting in a smoother surface, which influences the interaction and adhesion of the membranes. Gao et al. [43] report that longer process conditions reduced the Ra value from 25.3 nm to 0.4 nm due to the presence of hydrogen formation. These values agree with the current study, as seen in Table 5.

### 2.6. Cyclic Voltammetry (CV) and Multicycle CV Analysis

**Figure 10:**
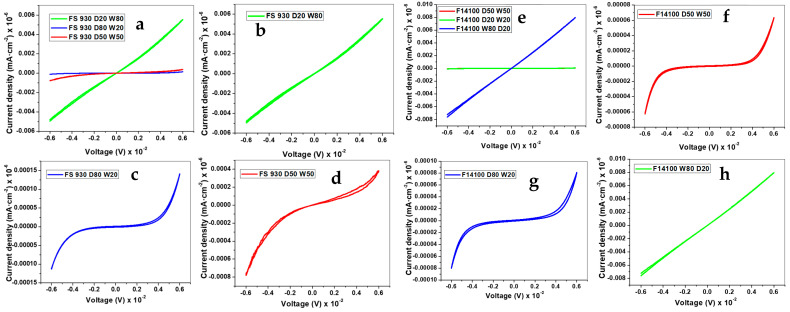
Cyclic Voltammetry of FS-930 (**a**–**d**) and F-14100 (**e**–**h**).

The membrane’s redox behavior includes details on its oxidation and reduction potentials, electron transfer kinetics, and the number of electrons involved [44]. The permselective qualities of electrode-supported ion-exchange polymer films for molecular-scale spectroscopic investigations of ionomer membranes were examined. Electrochemical experiments were conducted at standard laboratory temperature (21 ± 1 °C), as illustrated in Figure 10a–h, showing the CV and multi-cyclic CV of FS-930. The multicyclic voltammetry involved 20 consecutive scans at 0.6 mV, as illustrated in Figure 10a–d. The voltammograms support diffusion-limited transport of redox active probe ions within an electrode-supported ionomer film [45].

**Figure 11:**
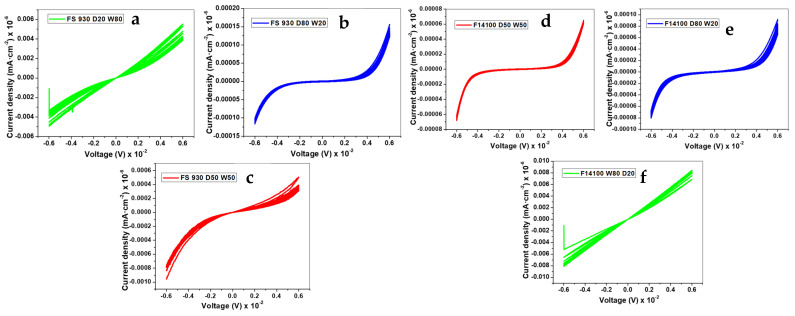
Multi-CV of FS-930 (**a**–**c**) and F-14100 (**d**–**f**).

Slightly curved and broad features were observed from the CV curves, suggesting efficient ionic transport with capacitive behavior. The multi-CV scans for FS-930 and F-14100 in Figure 11b,c,e,f exhibited stability with cycle number, which may be attributed to the activation of ionic clusters or structural restructuring upon repeated cycling. In FS-930 and F-14100 in Figure 10a,f, however, the current densities were significantly higher, and the voltammograms appeared more linear and less defined. This suggests that 80% DMSO and 20% water, and 50% of both solvents, promoted higher ion mobility and improved electrode–membrane contact for FS-930 and F-14100, whereas water limited the charge transport due to reduced solvent–polymer interactions. This is in accordance with the recent literature [46,47], with shorter-side-chain ionomers and better microstructures responding strongly to polar aprotic solvents in CV, achieving superior current densities when compared with their behavior in water.

### 2.7. EIS Nyquist Plot and Bode Plot Analysis

**Figure 12:**
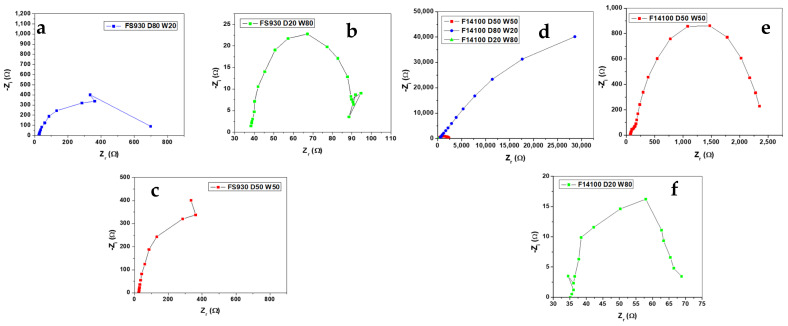
EIS Nyquist plot for F-14100 (**a**–**c**) and EIS Nyquist plot for FS-930 (**d**–**f**).

**Figure 13:**
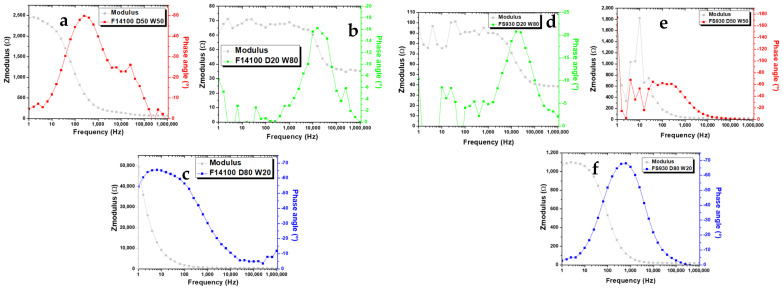
Bode plot of F-14100 (**a**–**c**) and FS-930 (**d**–**f**).

Figure 12a–f EIS curves for F-14100 and FS-930, while Table 6 shows the proton conductivity parameters obtained from the EIS at 0.6 mV for F-14100 and FS-930. EIS measurements shown in Figure 13c,d indicated that the F-14100 and FS-930 membranes exhibited a wider semicircle and charge-transfer resistance at the membrane–electrode interface. FS-930 D50-W50 and F-14100 D80-W20 membranes reveal a Warburg impedance, as seen in the linear segment incline at the terminus of the semicircle in the ideal capacitive region. This suggests the presence of a diffusion-controlled process at higher frequencies for both membranes, a phenomenon associated with typical Warburg presence in Nafion-based systems. On the other hand, the absence of Warburg impedance in FS-930 D80-W20, FS-930 D20-W80, F-14100 D20-W80, and F-14100 D50-W50 implies a predominance of charge-transfer resistance over diffusion-related processes [48]. The ionic conductivity of D80-W20 in both membranes was higher, which increased due to high DMSO content, when compared with other solvent ratios. The highest ionic conductivities of 1.74 × 10^−3^ and 1.38 × 10^−5^ S cm^−1^ were observed for D80-W20 in both membranes. Also, FS-930 D50 W50 has 1.38 × 10^−5^ S cm^−1^ ionic conductivity. This implies that proton conductivity is influenced by the DMSO content.

Figure 13a–c displays the Bode plot for F-14100, while Figure 13d–f presents the plot for FS-930 and F-14100. The Bode phase plot effectively illustrates the membranes’ primary electrical activity across a specific frequency range, facilitating visualization of electron diffusion processes. These observations were evidenced by the Bode plot’s frequency-dependent phase angle and impedance magnitude. DMSO-rich treated membranes exhibited a shift in phase-angle peaks to lower frequencies, indicating longer relaxation time constants and enhanced segmental motion within the polymer matrix, correlating with more open ion channels and increased ionic conductivity. Conversely, water-rich treated membranes displayed unchanged or slightly elevated phase angles, consistent with slower charge transfer and denser hydration layers.

### 2.8. Linear Sweep Voltammetry (LSV) Analysis

**Figure 14:**
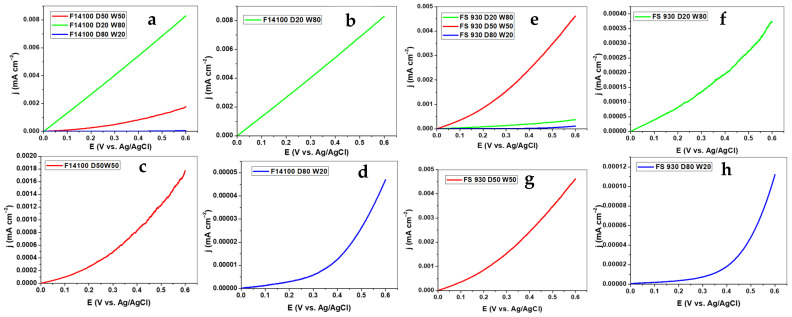
LSV of F-14100 (**a**–**d**) and FS-930 (**e**–**h**).

LSV was utilized to measure the current produced by linearly sweeping the potential applied to a working electrode between 0.1 and 0.6 mV. Figure 14a–h shows the LSV of F-14100 and the FS-930 curve of current potential activities of the membranes. A positive slope was observed for most membranes apart from the for 20% DMSO and 80% F-14100 membrane, with a straight line with increasing potential. FS-930 50% membrane showed a superior current at 0.0045 A compared to the others. These slopes could point to negligence of an internal electronic shortage, which hinders the charge transfer processes at the electrode–electrolyte interfaces, a major function of the membrane. The enhanced performance of the membrane can be attributed to and achieved due to factors such as membrane thickness, which ultimately influence the physical separation for anodic and cathodic reactions, leading to enhanced proton transport.

## 3. Materials and Methods

### 3.1. Materials and Methods

All chemical reagents used were of high purity and sourced from Sigma Aldrich (Johannesburg, South Africa). The Fumapem F-14100 waS a 20 cm × 30 cm non-reinforced PFSA/PTFE cation exchange membrane (CEM), while the Fumapem FS-930 was available in sizes of 10 cm × 10 cm or 20 cm × 30 cm. Membrane pieces of approximately 1 cm, supplied by Isondo Precious Metals, were subjected to dissolution in traditional solvents such as dimethyl sulfoxide (DMSO) and water. Temperature variation was monitored using a 305 mm glass mercury thermometer, while a Benchmark magnetic stirring hotplate ensured uniform mixing. Solvent volumes were measured with a graduated cylinder, and the mixed solution was contained in a 250 mL Erlenmeyer flask sealed with parafilm^®^ M All-Purpose Laboratory Film from Sigma Aldrich (Johannesburg, South Africa).

Ionomer membrane, specifically F-14100 and FS-930, was utilized as the membrane sample. These solvents were chosen to represent widely used aprotic and alcohol-based solvent systems pertinent to fuel cell applications, specifically in membrane reconditioning and ionomer dispersion. Based on average concentrations reported in ionomer dispersion and Nafion–solvent interaction experiments, a uniform membrane-to-solvent ratio was applied to all samples to guarantee compatibility. In order to promote polymer-solvent interaction and prevent heat decomposition of the membrane structure, a moderate and commonly utilized treatment temperature of 60 °C was used.

### 3.2. Selection of Solvents

A variety of solvent systems was prepared to explore the dissolution of membranes in them. For example, 80 mL of DMSO and 20 mL of H_2_O were combined to create 80% DMSO and 20% water, with a total volume of 50 mL. The same procedure was used to prepare other mixed solvents, 20% DMSO/80% H_2_O, and 50% DMSO/50% H_2_O, which were labelled D80:W20, D50:W50, and D20:W80, respectively. A similar approach was followed for the FS-930 membrane; labelled as D80:W20, D50:W50, and D20:W80, respectively.

### 3.3. Preparation of Mixed Solvents

The solvent ratio in the mixture varies based on the membrane type and the desired dissolution effect, with blends made from DMSO and water depending on the membrane’s solubility. Common preparation ratios included 50:50, 80:20, and 20:80 of the two solvents.

### 3.4. Immersion of the Membrane into the Solvents

The FS-930 and F-14100 membranes were cut into 1 cm sections and placed in mixed solvent systems, which were heated to around 60 °C and stirred at 300 rpm for 48 h to ensure uniform dissolution. Complete submersion was crucial for effective solvent penetration and homogeneous dissolution, with periodic observations made to track progress.

### 3.5. Post-Dissolution Procedure

After cooling, the solution was filtered through a 55 mm diameter Grade 1 Whatman TM filter paper to remove insoluble materials. In a 10 cm diameter Petri dish, a solution was poured and placed in an 80 °C drying oven for 24 h to evaporate solvents completely. Afterwards, deionized water was added to the dish, causing the PEM to swell and separate from the bottom. The membranes were dried in an oven for 12 h prior to conducting replicate measurements as per the experimental procedures [49,50,51].

### 3.6. Water Uptake Analysis

The water uptake analysis was achieved through a gravimetric approach by comparing the weight of the membrane before and after soaking in water. This assessment is vital due to the fact that water plays a major role in aiding proton movement through the membrane [51].

The percentage of water uptake for each membrane was then determined using Equation (1).

### 3.7. Characterization

Fourier Transform Infrared Spectroscopy (FTIR) analysis was conducted using a PerkinElmer Spectrum Two spectrometer (PerkinElmer, Waltham, MA, USA). Solid and thin-film materials were directly measured with the device’s attenuated total reflectance (ATR) accessory, which eliminated the need for extensive preprocessing. The machine included a lithium tantalate (LiTaO_3_) detector for high sensitivity, with a resolution of up to 1 cm^−1^ and a typical spectral range of 4000–400 cm^−1^. This arrangement allowed the tracking of chemical bonding, the identification of functional groups following membrane disintegration, and the detection of structural changes in the materials. X-ray diffraction (XRD) observations were made using a Bruker D8 Advance diffractometer (Cambridge, UK) equipped with a Cu Kα radiation source (λ = 1.5406 Å) operating at 40 kV and 40 mA. The apparatus included a goniometer in θ–2θ geometry and a Lynx Eye position-sensitive detector to produce high-resolution diffraction patterns. Data were collected over a 2θ range of 5–80° using a step size of 0.02° and a scan speed set for the optimal signal-to-noise ratio. This arrangement enabled phase identification, crystallinity analysis, and the computation of the sample lattice parameters. The surface morphology and microstructural characteristics of the sample were examined using scanning electron microscopy (SEM) with a JEOL JSM-IT300 microscope (Hitachi, Munich, Germany). High-resolution imaging at various magnifications was made possible by the device’s ability to accelerate voltages between 0.5 and 30 kV, which was powered by a tungsten electron source. Secondary electron (SE) and backscattered electron (BSE) detectors (Hitachi, Munich, Germany) produced topographical and compositional differences, respectively. By measuring the membranes’ impedance at various frequencies after dissolution, electrochemical impedance spectroscopy (EIS) examined the electrical characteristics of the membranes. The interfacial charge transport activities were evaluated using EIS. EIS Nyquist plots and bode plots were created using an impedance analyzer (Gamry 10101E Galvanostat/Potansiyostat/ZRA Reference 3000, Gamry Instruments, Warminster, PA, USA) that operated in the frequency range of 0.6 mV and 1.00 Hz–1.00 MHz. In this work, we employed the standard three-electrode setup for the EIS test, which included a working electrode, a saturated calomel reference electrode, and a Pt counter electrode. The redox behavior of the membrane, including its oxidation and reduction potentials, the kinetics of electron transfer, and the quantity of electrons involved, was revealed using cyclic voltammetry (CV). Four-probe electrode instruments (Gamry 10101E Galvanostat/Potansiyostat/ZRA Reference 3000) were used in a three-electrode configuration for the EIS test. A working electrode (graphene), a saturated calomel reference electrode, a Pt counter electrode, and an electrolyte consisting of a 0.2 M aqueous solution of H_2_SO_4_ was used. Using the identical Gamry apparatus in a three-electrode setup, cyclic voltammetry (CV) and multicyclic voltammetry were recorded with a potential window of –5.0 V to 5.0 V, a potential step of 2.0 mV, and a scan rate of 100 mV s^−1^. This test condition was used to identify the electrochemical and structural stability of the membrane/electrode system under extreme conditions from −5 V to +5 V. The current that arises from sweeping the voltage applied to a working electrode linearly across a range of 0.1 mV to 0.6 mV was measured using linear sweep voltammetry (LSV). Raman spectroscopy was performed using a Renishaw inVia Reflex Raman microscope (WITec GmbH, Ulm, Germany) to investigate the vibrational properties of the sample. The apparatus featured a 532 nm excitation laser, a confocal optical microscope for precise focusing, and a high-sensitivity CCD detector for signal capture. Spectra were collected in the 100–4000 cm^−1^ range with a spectral resolution of roughly 1 cm^−1^. The system included a notch filter to remove Rayleigh scattering and provide accurate Raman shift detection. This arrangement enabled the identification of functional groups, crystallinity studies, and the detection of structural or chemical changes in the materials. The hydrophobicity of the membrane was assessed by water uptake at intervals of 24, 48, 72, and 10 days. AFM and Raman spectroscopic analyses were performed using a WITec Alpha 300 RA system (WITec GmbH, Ulm, Germany) equipped with a confocal Raman–AFM module, enabling simultaneous acquisition of topographical and chemical information from identical sample regions. The surface of the samples was scanned in intermittent contact mode, employing force modulation cantilevers, with a resonance frequency of 75 kHz and a spring constant of 2.8 N/m.

## 4. Conclusions

This study showed that the dissolution of F-14100 and FS-930 membranes in mixed water and DMSO solvents at different ratios (20:80, 50:50, 80:20) at 48 h and a temperature of 60 °C significantly affected their structural and morphological properties. Significant changes in membrane morphology due to solvent content affected polymer swelling and dissolution rates. F-14100 showed higher sensitivity to solvent polarity and ionic content compared to FS-930. Solvent interactions led to a transformation from dense to porous structures, indicating a fundamental role in polymer chain rearrangement. FTIR, SEM, and electrochemical analyses highlighted discrepancies in molecular organization, with F-14100 experiencing more significant changes upon dissolution. Increased DMSO content strengthened solute–solvent interactions and influenced solvation behavior, with F-14100 exhibiting limited solubility in water-rich mixtures and better dissolution in DMSO. XRD analysis confirmed that both membranes were semi-crystalline; however, F-14100 demonstrated better crystallite size. The morphological differences revealed FS-930’s denser structure versus F-14100’s higher porosity, indicating that solvent composition significantly affects membrane characteristics. Electrochemical analysis indicated that redox behavior was influenced by transport mechanisms, with F-14100 showing higher charge-transfer resistance due to its thicker structure. DMSO-rich conditions improved ionic conductivity, while water-rich environments slowed charge transfer. Subtle electrochemical behavior differences between the membranes were observed, with F-14100 performing better than FS-930. The final take from this study was that solvent polarity and ratio have impacts on structural, morphological and ionic conductivity of both ionomer membranes, which can enhance their performance in fuel cells. This therefore suggests that future study on both membranes could benefit from combining other materials with them as a potential approach to improve fuel cell performance and other related technologies.

## Figures and Tables

**Table 1:** Raman spectrum vibrational modes of FS-930 and F-14100.

Stretching Bands	Vibrational Description	FS-930 (cm^−1^)	F-14100 (cm^−1^)
ν_s_(C–S) [cm^−1^]	linkage between –SO_3_H	1090 ± 0.23	1061 ± 0.03–1090 ± 0.03
ν_s_(C–F)_bb_ [cm^−1^]	Segments of CF_2_–CF_2_	1284 ± 0.08	1284 ± 0.04
ν_s_(C–F)_sc_ [cm^−1^]	CF_3_ and CF_2_ groups	1190 ± 0.07–1181 ± 0.07	1190 ± 0.02–1198 ± 0.02
ν_s_(C–O–C) [cm^−1^]	–O–CF_2_–CF_2_–O– (side chain)	1010 ± 0.05–1023 ± 0.05	980 ± 0.06–1002 ± 0.06
ν_s_(C–H) [cm^−1^]		626 ± 0.13	626 ± 0.01–634 ± 0.01

**Table 2:** Water uptake of FS-930 and F-14100 at different time intervals.

Time	Membrane Type	Mass of Dry Membrane (g)	Mass of Wet Membrane (g)	Water Uptake Percentage (%)
24 h	FS-930	0.0234 ± 0.34	0.0259 ± 0.04	10.68 ± 0.38
	F-14100	0.0412 ± 0.04	0.0488 ± 0.03	18.45 ± 0.07
48 h	FS-930	0.0383 ± 0.01	0.0437 ± 0.33	14.09 ± 0.32
	F-14100	0.0418 ± 0.05	0.0505 ± 0.01	20.81 ± 0.06
72 h	FS-930	0.0389 ± 0.43	0.0451 ± 0.03	15.94 ± 0.44
	F-14100	0.0425 ± 0.02	0.0540 ± 0.37	27.05 ± 0.35
10 days	FS-930	0.0394 ± 0.01	0.0465 ± 0.02	18.02 ± 0.04
	F-14100	0.0431 ± 0.03	0.0560 ± 0.04	29.93 ± 0.07

**Table 3:** XRD parameters for F-14100 and FS-930 membranes.

Membrane	Peak Position 2θ (°)	Calculated d-Spacing (Å)	FWHM (°)	Muller Index (hkl)	Crystallite Size (nm)
Blank	18.44 40.12	4.81 2.25	0.44 0.52	110/200 210	18.7 17.68
F-14100 D50 E50	18.53 40.24	4.79 2.24	0.44 0.56	110/200 210	18.7 17.69
F-14100 D80 E20	18.56 40.21	4.77 2.23	0.43 0.58	110/200 210	19.2 17.69
F-14100 W80 D20	18.68 40.37	4.78 2.24	0.46 0.50	110/200 210	17.8 17.69
Blank	18.13 39.60	4.89 2.28	0.46 0.53	110/200 210	18.1 17.65
FS-930 W50 D50	18.14 39.82	4.87 2.27	0.46 0.56	110/200 210	18.1 17.66
FS-930 D80 W20	18.22 39.79	4.87 2.27	0.48 0.57	110/200 210	18.3 17.66
FS-930 D20 W80	18.20 39.92	4.84 2.26	0.48 0.52	110/200 210	17.3 17.67

**Table 4:** Summary of Membranes elemental composition by EDX (* artifact peak).

Sample Name	Element Formula	Mass Percentage (%)	Atom Percentage (%)
(a) FS-930 (D80: W20)	C*	15.63 ± 0.20	20.06 ± 0.20
	O*	80.88 ± 0.58	77.96 ± 0.58
	F*	0.90 ± 6.22	0.73 ± 6.22
	S*	2.60 ± 0.16	1.25 ± 0.16
(b) FS-930 (D50: W50)	C*	16.25 ± 0.20	20.65 ± 0.20
	O*	82.47 ± 0.61	78.69 ± 0.61
	F*	0.14 ± 7.13	0.11 ± 7.13
	S*	1.15 ± 0.15	0.55 ± 0.15
(c) FS-930 (W80: D20)	C*	14.81 ± 0.20	18.87 ± 0.20
	O*	82.97 ± 0.61	79.34 ± 0.61
	F*	2.22 ± 7.59	1.79 ± 7.59
(d) F-14100 (D80: W20)	C*	18.67 ± 0.24	23.47 ± 0.24
	O*	80.90 ± 0.73	76.33 ± 0.73
	S*	0.43 ± 0.15	0.20 ± 0.15
(e) F-14100 (D50: W50)	C*	16.36 ± 0.21	20.70 ± 0.21
	O*	83.28 ± 0.64	79.13 ± 0.64
	S*	0.37 ± 0.15	0.17 ± 0.15
(f) F-14100 (D20: W80)	C*	16.87 ± 0.20	21.32 ± 0.20
	O*	82.68 ± 0.65	78.46 ± 0.65
	S*	0.46 ± 0.15	0.22 ± 0.15

**Table 5:** Summary of AFM 3D surface roughness of F-14100 and FS-930 membranes.

Sample	Average Roughness (μm)
F-14100 D50 E50	0.082 ± 0.03
F-14100 D80 E20	0.076 ± 0.03
F-14100 W80 D20	0.084 ± 0.01
FS-930 W80 D20	0.065 ± 0.02
FS-930 D80 W20	0.045 ± 0.04
FS-930 D50 W50	0.086 ± 0.02

**Table 6:** Proton conductivity parameters for 0.6 mV and 1.00 Hz- 1.00 MHz from the EIS for F-14100 and FS-930.

Sample	EIS Parameters		
	Rs (Ω)	R1 (Ω)	σ (mS cm^−1^)
F-14100 D50 E50	71.15 ± 0.24	75.29 ± 0.76	2.55 × 10^−7^
F-14100 D80 E20	7.47 ± 0.4	1.64 ± 0.5	1.74 × 10^−3^
F-14100 W80 D20	35.58 ± 0.54	31.70 ± 0.90	4.39 × 10^−7^
FS-930 W80 D20	37.15 ± 0.68	48.14 ± 0.49	4.44 × 10^−7^
FS-930 D80 W20	25.76 ± 0.25	593.56 ± 2.99	1.38 × 10^−5^
FS-930 D50 W50	25.76 ± 0.25	593.56 ± 2.99	1.38 × 10^−5^

## Data Availability

The data supporting the results of this investigation are included in the manuscript.

## Abbreviations

The following abbreviations are used in this manuscript:

ATR	Attenuated total reflectance
CEM	Cation exchange membrane
CRM	Confocal Raman microscopy
CV	Cyclic voltammetry
DMAc	Dimethylacetamide
DMF	Dimethylformamide
DMSO	Dimethyl sulfoxide
EDX	Energy dispersive X-ray spectroscopy
EIS	Electrochemical impedance spectroscopy
FTIR	Fourier transform infrared spectroscopy
HFP	Hexafluoropropylene
H_2_O	Water
HFPO	Hexafluoropropylene oxide
LSV	Linear sweep voltammetry
NMR	Nuclear magnetic resonance
PEM	Proton exchange membrane
PEMFCs	Proton exchange membrane fuel cells
PES	Polyethersulfone
PFPEs	Perfluoropolyethers
PFSA	Perfluorosulfonic acid
PS	Polysulfone
PTFE	Polytetrafluoroethylene
PVDF	Polyvinylidene fluoride
SE	Secondary electron
TFE	Tetrafluoroethylene
VDF	Vinylidene fluoride
XRD	X-ray diffraction
